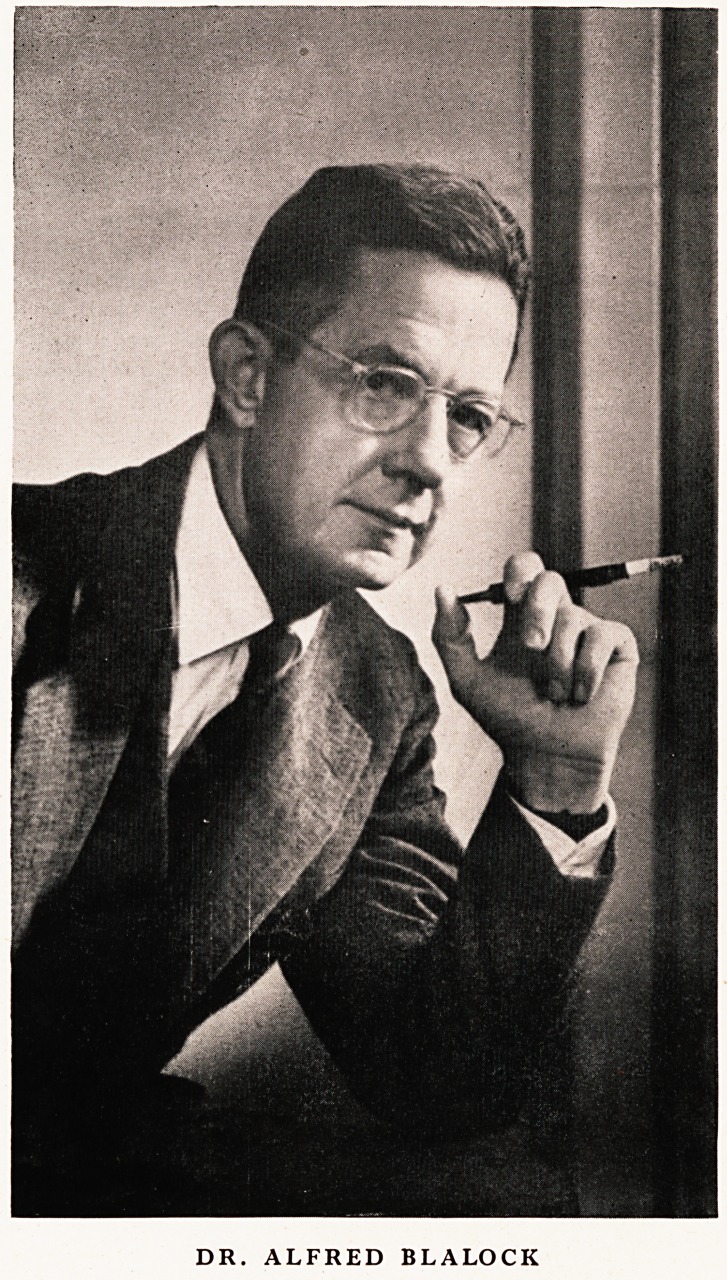# Visit of Dr. Alfred Blalock

**Published:** 1952-07

**Authors:** 


### VISIT OF DR. ALFRED BLALOCK

On May 6th, Bristol was honoured by a visit from Dr. Alfred Blalock, Professor of Surgery at the Johns Hopkins Hospital, Baltimore. Accompanying him was Dr. Glen Morrow, one of the Resident Surgeons of the hospital. They were met at Southampton by Professor Milnes Walker and Mr. Eric Nanson and were driven to Bristol in time for Dr. Blalock to deliver a lecture at mid-day in the Royal Fort. Dr. Blalock was introduced by the Vice-Chancellor to a crowded gathering of doctors, students and nurses.

Dr. Blalock is the third notable occupant of the Chair of Surgery at Johns Hopkins Hospital. First was William Stewart Halstead, the centenary of whose birth, celebrated by the Royal Society of Medicine on May 20th, was the reason for Dr. Blalock's visit to England. The second was Dean Lewis, and he was succeeded by Dr. Blalock who came to Hopkins from Vanderbilt University, Tennessee, in 1941. The world probably knows of Dr. Blalock as the originator of the " Blue Baby operation ", but he himself values more highly the work he did in the 'thirties on the fundamental pathology and treatment of shock, which is now classical. He stressed the part played by loss of blood volume in shock production and the vital importance of its adequate replacement. He was one of the first men to perform thymectomy for myasthenia gravis. In 1944 he performed the first subclavian to pulmonary artery anastomosis for Tetralogy of Fallot, now known as the Blalock-Taussig operation. The brilliance of this operation lies not so much in the technical details, but in the fact that he and Taussig appreciated that in Tetralogy of Fallot there was a diminished pulmonary blood flow which caused the patient's disability and was the point of attack for his relief. Since 1944, some seven hundred such operations have been performed at the Johns Hopkins Hospital. By this work Dr. Blalock has widened the field of cardiac surgery and in particular that of congenital heart disease, thereby enhancing the Halstead tradition and making Baltimore the leading cardiac surgical centre of the world.

As a teacher both of undergraduates and postgraduates he is pre-eminent, and the famous Friday noon clinics which he conducts, as in the days of Halstead, are attended with pleasure and profit by visitors to Baltimore. As a man Dr. Blalock has great charm of personality. A characteristic is his modesty in giving credit to his assistants rather than to himself.

After his lecture he had lunch with the senior registrars at the Bristol Royal Infirmary, and he and Dr. Morrow spent the afternoon seeing and discussing the work that was in progress in the Department of Surgery. In the evening he dined with some of the members of the surgical and medical staff.

In his lecture Dr. Blalock discussed the cardio-vascular problems which he and his associates have been treating at the Johns Hopkins Hospital. He stated that the results of sympathectomy for essential hypertension had been moderately successful. In wounds of the heart he advocated pericardial aspiration rather than open operation as the primary measure. He described three cases of

DR. ALFRED BLALOCK

saccular syphilitic aneurysm of the aorta treated by Dr. Henry Bahnson by excision of the aneurysm and repair of the aorta. He illustrated the excellent result to be obtained by lobectomy for congenital pulmonary arterio-venous aneurysm.

He divided surgery of the heart itself into two major divisions; for congenital lesions, and for acquired lesions.

*Congenital lesions.* He described the four-ligature method of closing a patent ductus arteriosus, and commented on the atypical large ductus associated with pulmonary hypertension and without a continuous machinery murmur. In the Johns Hopkins Hospital some 130 cases of coarctation of the aorta had been operated on and he illustrated one case in which an aplastic segment of aorta 5 cm. long had been resected and replaced by a homograft. The optimum time for an operation on coarctation of the aorta was five to fifteen years of age.

He reviewed the results of 691 cases of cyanotic heart disease, mainly cases of Tetralogy of Fallot, which had been operated on in his cardiac unit. In eighty-two per cent. the results had been good, in two per cent. fair and the mortality was fourteen per cent. He reviewed the type of stenosis in ninety-five cases of Tetralogy of Fallot from post-mortem examination of the heart and found that in sixty-three there was an infundibular stenosis and a valvular stenosis in only ten.

*Acquired Heart Disease.* He discussed surgery of mitral valvular stenosis, fifty cases having been operated on in his clinic. He gave the indications and contra-indications which fitted in closely with our own criteria in Bristol. There had been five deaths, chiefly due to cerebral embolism, which he regarded as the main hazard of the operation. The results he thought most encouraging.

He touched on the surgery of constrictive pericarditis and stressed the importance of clearing both ventricles, and thought that earlier operation could be undertaken. He mentioned the work of Beck on coronary arterial disease but doubted whether it was a sound surgical procedure.

The lecture was a masterly review of the subject, illustrated with beautiful lantern slides, and couched in such clear terms that those unfamiliar with cardiac surgery could follow it with ease.

## Figures and Tables

**Figure f1:**